# Supplementary material for: Development and Application of Automated Sandwich ELISA for Quantitating Residual dsRNA in mRNA Vaccines
Source: Vaccines (Basel). 2024 Aug 8;12(8):899. doi: 10.3390/vaccines12080899 (PMC11359411; doi:10.3390/vaccines12080899)
Supplement: Supplementary file 1 [file vaccines-12-00899-s001.zip › vaccines-3097740-supplementary.pdf]

Development and Application of Automated Sandwich ELISA for Quantitating Residual dsRNA in mRNA Vaccines

David A. Holland [1,*], Jillian Acevedo-Skrip [1], Joshua Barton [1], Rachel Thompson [1], Amy Bowman [1], Emily A. Dewar [2], Danielle V. Miller [2], Kaixi Zhao [2], Andrew R. Swartz [2] and John W. Loughney [1,*]

[1] Analytical Research & Development, Merck & Co., Inc., Rahway, NJ 07065, USA;
[2] Process Research and Development, Merck & Co., Inc., Rahway, NJ 07065, USA;
* Correspondence: david.holland1@merck.com (D.A.H.); john_loughney@merck.com (J.W.L.)

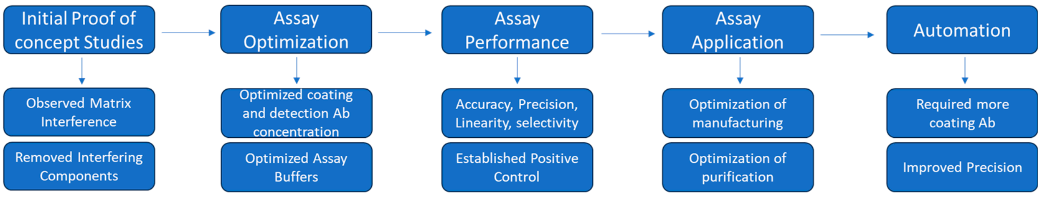

Figure S1: Block diagram representation of the study overview highlighting key information from each step.

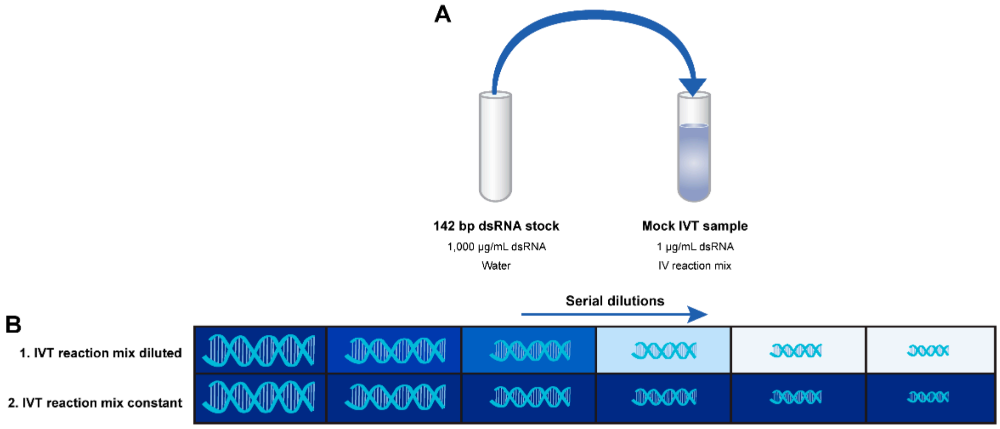

Figure S2: Schematic representation of the IVT mixture interference assay. A. A mock IVT sample was prepared by spiking 142 bp dsRNA into IVT reaction mixture to a target dsRNA concentration of 1000 ng/mL. B. The dsRNA ELISA serial dilution step was performed using either; 1. STE buffer, allowing the IVT reaction mixture to be diluted out over the serial dilutions; 2. STE buffer containing IVT matrix components, keeping the IVT matrix constant over the dilution series.

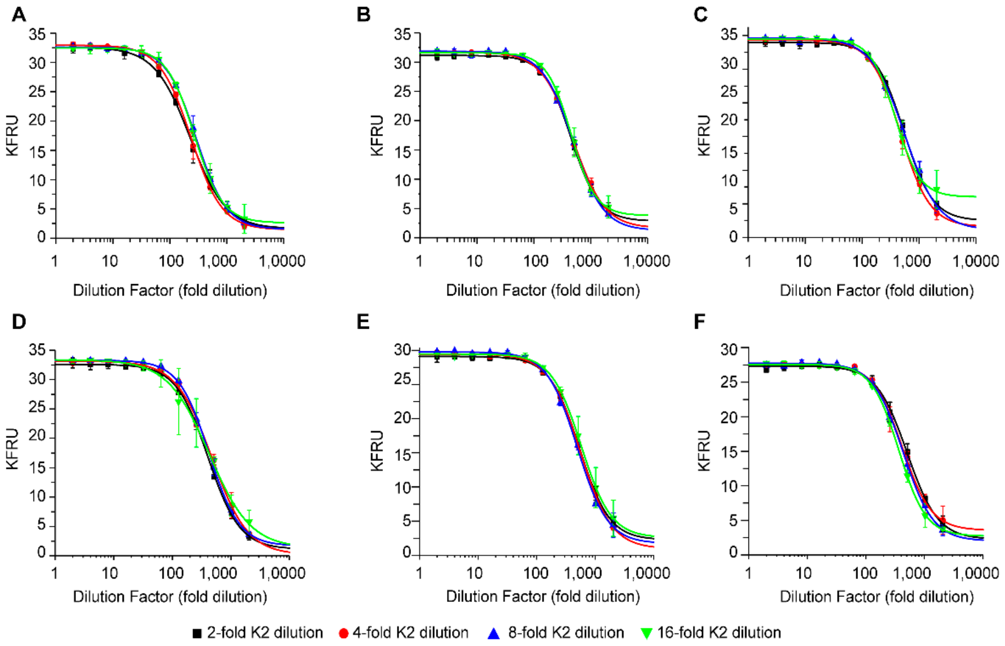

Figure S3: Optimization of coating antibody and detection antibody conditions. The dsRNA ELISA was performed using either 2 μg/mL (Panels A&D), 4 μg/mL (Panels B&E), or 8 μg/mL (Panels C&F) J2 capture antibody in 1X PBS (Panels A-C) or NH_4SO_4 (Panels D-F). For each coating condition, the assay was performed with either a 2-fold (black), 4-fold (red), 8-fold (blue) or 16-fold (green) dilution of K2 detection antibody. Each condition was measured in duplicate. Error bars represent 1 standard deviation of duplicate measurements.

Table S1: Nucleotide length of 7 mRNA constructs.

Construct	Length (nts)
Construct 1	1800
Construct 2	1800
Construct 3	1900
Construct 4	1900
Construct 5	1900
Construct 6	3800
Construct 7	4300

dsRNA contribution as an effect of IVT Reaction mixtures

To improve mRNA yield, 3 IVT additives were evaluated. The effect of these additives on the residual dsRNA concentration is listed in Supp. Figure 1. Additive 2 (green bar) had no effect on the residual dsRNA concentration relative to control (dark blue bar) whereas additives 1 and 3 (red and purple, respectively) induced a marginal decrease in residual dsRNA relative to control. A combination of all 3 additives (light blue), demonstrated a similar effect as additives 1 and 3 were used alone. Overall, these results suggest that the 3 additives evaluated as a part of this study had little to no effect on the residual dsRNA.

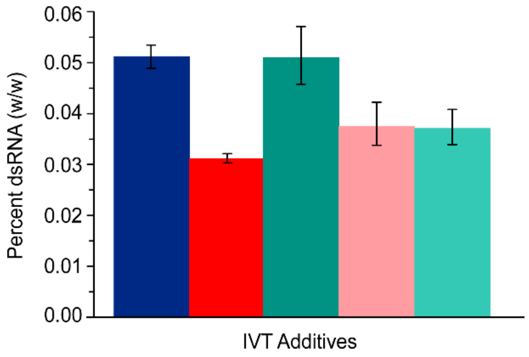

Figure S4: Effect of additive 1 (red), additive 2 (green) additive 3 (light red) or a combination of all three (light green) on the relative % dsRNA in IVT reaction products relative to the control (dark blue). Error bars represent 1 standard deviation of 3-4 dilutional replicates.

Optimization of Antibodies for Fully Automated ELISA

The dose-response curve of the 142 bp dsRNA RS was generated using plates coated with either 2 μg/mL J2 antibody (Supp. Figure 4A) or 4 μg/mL J2 antibody (Supp. Figure 4B), detection performed with either a 15-fold (black lines), 8-fold (red lines), 4-fold (green lines) or 2-fold (blue lines) dilution of K2 antibody and either 0.3 μg/mL (solid lines) or 0.6 μg/mL of conjugate Antibody (dashed lines). As seen in Supp. Figure 4, increasing the concentration of J2 coating antibody from 2 μg/mL to 4 μg/mL shifted the curve to the left and allowed for full production of the upper plateau. No trend was observed by increasing the concentration of either the K2 detection antibody or the conjugate antibody.

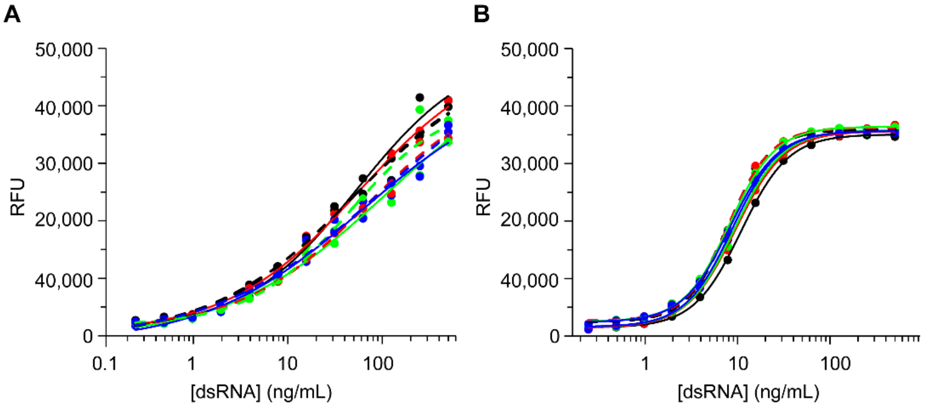

Figure S5: Optimization of coating antibody and detection antibody conditions in the fully automated ELISA. The dsRNA ELISA was performed using either 2 μg/mL (Panel A) or 4 μg/mL (Panel B), mouse anti-dsRNA J2 IgG for the coating antibody. For each coating antibody concentration, the assay was performed with either a 15-fold (black), 8-fold (red), 4-fold (green) or 2-fold (blue) dilution of mouse anti-dsRNA K2 IgM for the detection antibody and with either 0.3 μg/mL (solid lines) or 0.6 μg/mL (dashed lines) AP conjugated goat anti-mouse IgG. Each condition was measured in singlet and head-to-head (i.e. performed on the same day by the same analyst).

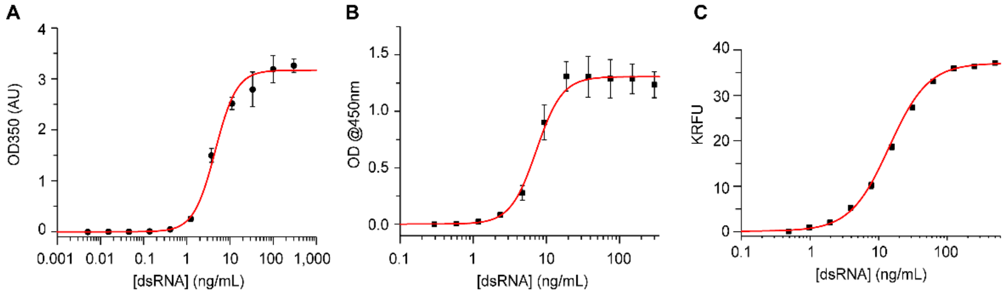

Figure S6: Representative dsRNA ELISA dose response curves generated using commercial kit 1 (A), commercial kit 2 (B) or in-house ELISA using bulk reagents (C). Error bars represent 1 standard deviation of triplicate measurements.

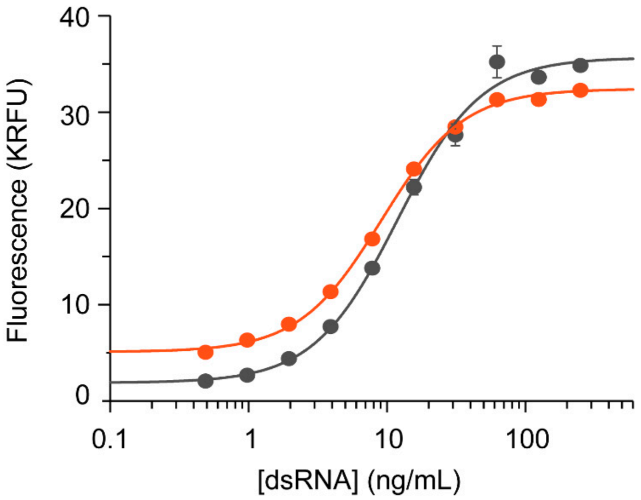

Figure S7: Representative dsRNA ELISA dose response curves generated using 142 bp dsRNA reference standard (black) and Poly I:C positive control (red). Curve of Poly I:C positive control generally lacks parallelism with the 142 bp dsRNA, consequently the concentration determined from the top dilution and the bottom dilution differ by over 300%. 9 replicate measurements of a 1 µg/mL solution of Poly I:C positive control performed on 3 separate days gave an apparent concentration of 3.0 ± 0.5 µg/mL.
